# From medication to meditation as treatment for chronic stress and utility of hair cortisol measurement: randomized clinical trial

**DOI:** 10.1192/j.eurpsy.2022.2283

**Published:** 2022-09-01

**Authors:** O. De Juan Viladegut, M. Bioque, L. Bueno Sanya, H. Andreu Gracia, L. Olivier Mayorga

**Affiliations:** Hospital Clínic de Barcelona, Psychiatry And Psychology, Barcelona, Spain

**Keywords:** Stress, Mindfulness, Cortisol, Anxiety

## Abstract

**Introduction:**

Stress is part of the daily life of many people, especially in Western countries. Recent studies have shown that cortisol has been listed as the primary hormone linked to stress. Currently, to measure cortisol values there are only tests that quantify it at a determined time point, without taking into account its variability and its changing pattern over time, depends on the circadian rhythm and other stress-related factors.

**Objectives:**

This randomized clinical trial of the Hospital Clínic de Barcelona proposes to study the utility of accumulated hair cortisol concentration (HCC) as a measure to correlate the levels of this hormone over time with the stress suffered by the patient.

**Methods:**

Patients are classified into two groups: a control group and an intervention group. In the second group, the participants will follow a mindfulness-based cognitive therapy (MBCT) with the aim of reducing stress and, consequently, cortisol levels.

**Results:**

The purpose of this study is to validate the utility of HCC in order to, retrospectively, obtain cortisol secretion curves as a measure of the level of stress of each individual and personalize the treatments. Simultaneously, we intend to present new perspectives for treatment in psychiatric disorders where stress predominates, such as generalized anxiety disorder (GAD) or major depression (MD), which are becoming increasingly important in our society.

**Conclusions:**

HCC contributes to the practice of personalized medicine as it allows us to detect cortisol exposure in the months prior to obtaining the capillary sample, and thus to draw the trend of this hormone over time.

**Disclosure:**

No significant relationships.

